# Parameters Identification of Rubber-like Hyperelastic Material Based on General Regression Neural Network

**DOI:** 10.3390/ma15113776

**Published:** 2022-05-25

**Authors:** Junling Hou, Xuan Lu, Kaining Zhang, Yidong Jing, Zhenjie Zhang, Junfeng You, Qun Li

**Affiliations:** 1 State Key Laboratory for Strength and Vibration of Mechanical Structures, School of Aerospace Engineering, Xi’an Jiaotong University, Xi’an 710049, China; junlhou@mail.xjtu.edu.cn (J.H.); 3120306013@stu.xjtu.edu.cn (X.L.); knzhang@stu.xjtu.edu.cn (K.Z.); zzj1998@stu.xjtu.edu.cn (Z.Z.); 2 Research Institute of Xi’an Jiaotong University, Hangzhou 311215, China; 3 Xi’an Jiaotong University Suzhou Institute, Suzhou 215123, China; 4 Xi’an Modern Chemistry Research Institute, Xi’an 710065, China; jyd204@sina.com; 5 The 41st Institute of the Forth Academy of CASC, Xi’an 710025, China; youjunfeng@126.com; 6 Solid Rocket Motor National Key Laboratory of Combustion Flow and Thermo-Structure, Xi’an 710025, China

**Keywords:** general regression neural network (GRNN), hyperelastic material model, parameters identification

## Abstract

In this study, we present a systematic scheme to identify the material parameters in constitutive model of hyperelastic materials such as rubber. This approach is proposed based on the combined use of general regression neural network, experimental data and finite element analysis. In detail, the finite element analysis is carried out to provide the learning samples of GRNN model, while the results observed from the uniaxial tensile test is set as the target value of GRNN model. A problem involving parameters identification of silicone rubber material is described for validation. The results show that the proposed GRNN-based approach has the characteristics of high universality and good precision, and can be extended to parameters identification of complex rubber-like hyperelastic material constitutive.

## 1. Introduction

Hyperelastic materials have advantages of high elasticity, shock resistance, wearability and many other excellent properties, and can be capable of undergoing large deformation. In real engineering application, many materials show hyperelastic properties, such as rubbers, gels, insulation of solid rocket motor and soft tissue (arteries, muscles, and skin). During the past years, from the perspective of studying the mechanical behavior of hyperelastic materials, some scholars focused on how to use theoretical models to predict and describe the experimental phenomenon [1,2,3]. In addition, technological workers also paid attention to the numerical simulations of the complex response of devices with hyperleastic property based on specific experimental data [4,5]. It can be claimed that the establishment of hyperelastic materials constitutive model is a key problem of common concern to researchers. 

Research shows that the hyperelastic material has the characteristics of nonlinear, large deformation, and its constitutive properties entirely depend on its strain energy function [6,7]. Following this idea, several hyperelastic constitutive models have been proposed to provide a suitable strain energy density for a given hyperelastic material through appropriate theories and methods, such as the Mooney–Rivlin model [8,9], Ogden model [10], Gent model [11,12], Gent–Thomas model [13] and Carroll model [14], etc. According to the characteristics of deformation, these models can be roughly divided into four categories, including constitutive models of compressible, incompressible, full deformation range and various deformation modes. Among them, the study on the strain energy density of incompressible hyperelastic models become a basic and important work since the incompressible conditions greatly simplify the theoretical research and engineering application of hyperelastic material properties [15,16], and for the further application of rubber-like hyperelastic materials or structures, there is a recognized need for further investigation of the hyperelastic model [17,18]. Thereby, we focus on the parameter identification of the incompressible constitutive model in the present study. 

At the present stage, the common known approaches for determining hyperelastic material parameters, namely the strain energy density function coefficient, mainly include experiments [19,20,21], numerical calculation [22,23] and artificial intelligence methods [24,25]. In particular, artificial intelligence methods can predict the related parameters, which cannot be obtained directly or are difficult to obtain through experiment and simulation, and have received widespread attention in recent years. They are capable of establishing the relationship among variables based on the existing data, which is different from the traditional mechanical analysis method. Artificial intelligence has shown its advantages in the prediction of mechanical parameters, optimization of mechanical models, health monitoring and many other aspects [26,27,28,29,30], and has become the focus of many researchers and the trend of development. For instance, Nair et al. [30] inversed the constitutive parameters of soft biological materials based on genetic algorithm, numerical simulation and experimental testing deformation. By using the back propagation neural network optimized with genetic algorithm, Li et al. [31] predicted the values of dynamic stiffness and loss factor varied with the different frequency and discussed the frequency dependence of rubber bushing. 

As one kind of artificial intelligence-based approach, GRNN is a special form of radial basis function neural network [32,33,34,35,36], and has been widely applied to many fields [37,38,39]. Compared with the current popular feedforward neural network, it has a number of advantages. Firstly, the network structure of GRNN is relatively simple. Except the input and output layers, only two hidden layers, mode layer and summation layer are included generally, and the number of hidden units in the mode layer is equal to the number of learning samples. Otherwise, the training of the GRNN model is undemanding. As soon as the learning samples pass through the hidden layer, the training of the GRNN model is immediately completed, which does not require very long training time and high computational cost. In addition, due to its simple network structure, there is no need to estimate the numbers of hidden layers and hidden units. Only one free parameter, i.e., the smoothing factor of radial basis function, is required for GRNN learning, for which the optimization value can be easily obtained by the cross validation method. Moreover, it is worth emphasizing that the results of the GRNN calculation have good global convergence, and are better than the results of standard feedforward neural networks [39], which often fail to achieve global convergence and stop at local convergence.

The main aim of this paper is to develop a convenient and effective GRNN-based approach to identify the model parameters of hyperelastic material. This GRNN-based approach consists of experiments, numerical simulations, and GRNN learning, which are relatively easy to operate and obtain. The remainder of this paper is organized as follows. The theoretical basis and architecture of GRNN are explained in Section 2. The application of the GRNN-based approach in determining material parameters is performed in Section 3, including a brief introduction of the hyperelastic model, a prediction scheme of M-R model parameters based on GRNN, uniaxial testing and finite element analysis, and an example of validation using rubber material (see Section 3.3). Details on the prediction results of model parameters are contained and discussed in Section 4. Finally, the paper is concluded with remarks and a discussion of the proposed GRNN-based approach.

## 2. GRNN Method

### 2.1. Theoretical Basis of GRNN

On the basis of kernel non-parametric regression, GRNN has taken the sample data as a posteriori probability to implement non-parametric estimation, and the correlation density function between a dependent variable and an independent variable is calculated from the learning samples, so as to obtain the regression value of a dependent variable relative to an independent variable. A detailed description of the GRNN model is presented as below. 

Assuming that ***x*** and *y* are the input vector and output variable of the sample data, respectively, when the observed value of ***x*** is set as ***x***_0_, i.e., the commonly known target value, then the regression value *y* with respect to the input vector ***x*** is obtained by

(1) $$y\left(x_{0}\right)=\frac{\int_{-\infty }^{0}yf\left(x_{0},y\right)dy}{\int_{-\infty }^{0}f\left(x_{0},y\right)dy},$$

Here, *f*(***x***_0_, *y*) represents the probability density function. Using Parzen non-parametric estimation [40], the Gaussian kernel function is selected as the kernel function, and then the density function *f*(***X***, *Y*) can be calculated based on the sample data ${\left\{X_{i},\;Y_{i}\right\}}_{i=1}^{n}$, shown as below

(2) $$f\left(X,Y\right)=\frac{1}{{\left(2\pi \right)}^{\left(\frac{l+1}{2}\right)}k^{\left(l+1\right)}n}\cdot \sum_{i=1}^{n}\exp\left[-\frac{{\left(X-X_{i}\right)}^{T}\left(X-X_{i}\right)}{2k^{2}}\right]\cdot \exp\left[-\frac{{\left(Y-Y_{i}\right)}^{2}}{2k^{2}}\right],$$

where *k*, *n*, and *l* represent the smoothing factor, numbers of learning samples, and the dimension of the input vector, respectively.

Combining Equations (1) and (2), we obtain

(3) $$Y\left(X_{0}\right)=\frac{\sum_{i=1}^{n}Y_{i}\exp\left[-\frac{{\left(X-X_{i}\right)}^{T}\left(X-X_{i}\right)}{2k^{2}}\right]}{\sum_{i=1}^{n}\exp\left[-\frac{{\left(X-X_{i}\right)}^{T}\left(X-X_{i}\right)}{2k^{2}}\right]},$$

Equation (3) is the final expression of the GRNN model. It is worth noting that the value of the smoothing factor *k* has a great impact on the performance of the neural network, which needs to be optimized. As the smoothing factor approaches zero, the predicted value will be very close to the sample value, resulting in an over-fitting phenomenon. However, if the smoothing factor is very large, the predicted value will approximate the average of all samples.

### 2.2. Architecture of GRNN

GRNN is a kind of radial basis function (RBF) neural network, which has been proposed by Specht [32]. It is a feedforward neural network, which means the data feedback process is not contained and each layer is passed through forward computation. Different from the traditional three-layer architecture of RBF neural network, i.e., input, hidden, and output layers, the architecture of GRNN has four main layers. An additional summation layer is contained in hidden layer of GRNN, while the input and output vectors remain unchanged. In the following, the feature of each layer in the GRNN model is described briefly.

(1) Input layer

The number of neurons at the input layer is equal to the dimension *l* of the input vector of the learning sample. Each neuron is a simple and individual unit, which can directly transfer input variables to the pattern layer.

(2) Pattern layer

The number of neurons at the pattern layer is the same as the number of learning samples *n*, that is, each neuron corresponds to a specific learning sample. The transfer function of neuron at the pattern layer is expressed by

(4) $$P_{i}=\exp\left[-\frac{{\left(X_{0}-X_{i}\right)}^{T}\left(X_{0}-X_{i}\right)}{2k^{2}}\right],i=1,2\ldots ,n$$

(3) Summation layer

The summation layer uses two types of neurons for summation. The first kind of neuron considers the arithmetic sum of all neurons at the pattern layer. At this time, the link weight between the pattern layer and each neuron is defined as one, and the transfer function is

(5) $$S_{D}=\sum_{i=1}^{n}P_{i}$$

The second type of neuron carries out weighted summation of all neurons at the pattern layer. Specifically, the connection weight between the *i*th neuron at the pattern layer and the *j*th neuron at the summation layer is the *j*th element of output variable *Y_j_* in the *i*th learning sample, the corresponding transfer function is

(6) $$S_{Nj}=\sum_{i=1}^{n}Y_{ij}P_{i}$$

(4) Output layer

Similarly, the number of neurons at the output layer is equal to the dimension of the output vector in the learning sample. The value of *j*th neuron corresponds to the *j*th element of the predicted *Y*(***X***), which is given as

(7) $$Y_{j}=\frac{S_{Nj}}{S_{D}}$$

## 3. Application of GRNN for Determining the Hyperelastic Model Parameters

### 3.1. Hyperelastic Material Model

Hyperelastic material is a kind of nonlinear elastic material with large deformation capacity, and its material mechanical characteristics are completely described by its strain energy function [41]. Thus, for the investigation of hyperelastic material, the first key is to find appropriate theories and methods to determine its strain energy function. 

In the past, several hyperelastic constitutive models have been established [8,10,14]. Among them, the phenomenological constitutive models based on the continuum mechanics have been widely used for numerical calculations and implemented through software, such as ABAQUS. The strain energy function *W* of the phenomenological constitutive model is usually a function of invariants of deformation tensors (*I*_1_, *I*_2_ and *I*_3_) or principal extension ratios (*λ*_1_, *λ*_2_ and *λ*_3_), that is, *W* = *W*(*I*_1_, *I*_2_, *I*_3_) or *W* = *W*(*λ*_1_, *λ*_2_, *λ*_3_).

It has been found that most hyperelastic materials have features of very small volume change [14], and thus the strain energy functions with incompressible conditions have been developed by some scholars, such as in the Mooney–Rivlin model [8,9] and the Ogden model [14], etc. These models have their own advantages for different application cases. For instance, the Mooney–Rivlin model is applicable to analyze the small deformation and medium large deformation problems of hyperelastic materials. It is also the most widely used model in describing isotropic hyperelastic material behavior. The Ogden model can reflect the mechanical behavior under multi-axial states, such as uniaxial, biaxial and planar shear loads.

In the present study, three strain energy functions, including the Mooney–Rivlin (abbreviated as M–R) model and the polynomial model with *N* = 2, and the Ogden model with *N* = 3, which can provide an excellent simulation of hyperelastic mechanical behavior, are considered for the purpose of validating the accuracy and effectiveness of a GRNN-based approach. The definitions of strain energy for these three models are given below.

- The M–R model can be defined by two parameters, *C*_10_ and *C*_01_, shown as below,

(8) $$W_{1}=C_{10}(I_{1}-3)+C_{01}(I_{2}-3),$$

where *C*_10_ and *C*_01_ are model parameters which need to be determined.

- The formulation of polynomial model (*N* = 2) is given by,

(9) $$W_{2}\left(I_{1},I_{2}\right)=\sum_{i+j=1}^{2}C_{ij}{\left(I_{1}-3\right)}^{i}{\left(I_{2}-3\right)}^{j}$$

where *C_ij_* is the corresponding model parameter, including *C*_01_, *C*_10_, *C*_20_, *C*_11_ and *C*_02_.

- Based on the principal extension ratio, the strain energy of the Ogden model can be defined as,

(10) $$W=\sum_{i=1}^{N}\frac{\mu_{i}}{\alpha_{i}}\left({\bar{\lambda }}_{1}^{\alpha_{i}}+{\bar{\lambda }}_{2}^{\alpha_{i}}+{\bar{\lambda }}_{3}^{\alpha_{i}}-3\right)+\sum_{i=1}^{N}\frac{1}{D_{i}}{\left(J-1\right)}^{2i}$$

where *N* is the order the model (N = 3 in the present study). *μ_i_* and *α_i_* are material parameters related to temperature. *D_i_* = 0 for incompressible strain energy, while *J* is the elastic volume ratio. Thereby, six parameters, i.e., *μ*_1_, *α*_1_, *μ*_2_, *α*_2_, *μ*_3_, and *α*_3_, are the unknown parameters for the definition of the Ogden model.

### 3.2. The Parameter Identification Methodology for a Hyperelastic Model Based on Finite Element Analysis, Experiment and GRNN

**Figure 1:**
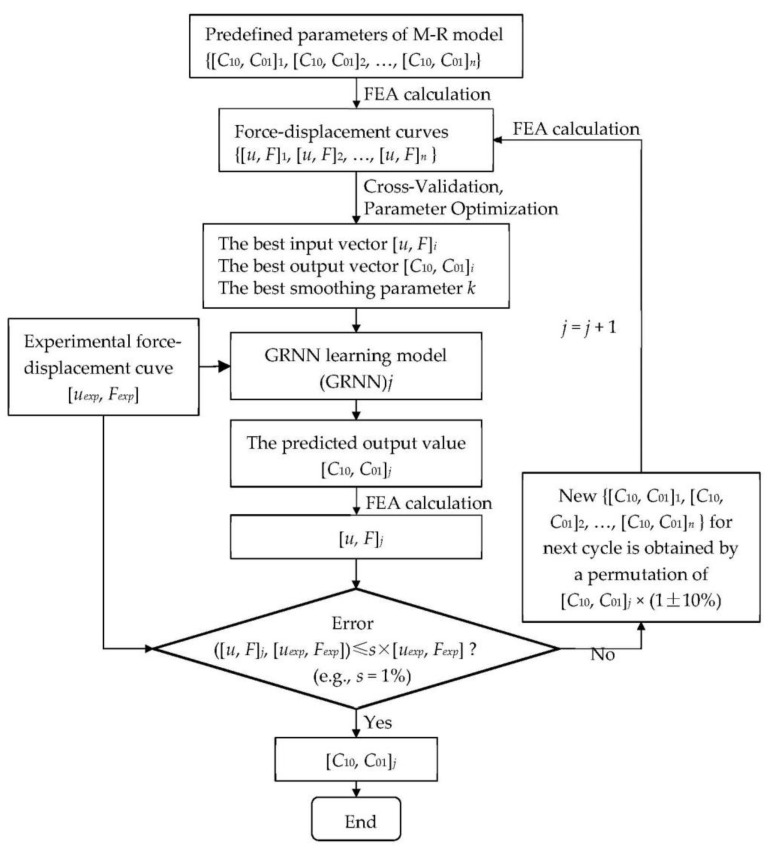
The scheme of the topological structure of a GRNN-based approach for the prediction of model parameters of M–R model. Nomenclature: *u* displacement; *F* reaction force; *n* number of learning samples; *exp* experimental results; *j* the cycle number of the GRNN model; *s* the given accuracy requirement.

See Figure 1, the parameter identification methodology for the M–R model based on finite element analysis (FEA), experiment and GRNN learning is presented as follows:

(a) Prepare the target values of the GRNN model. For this case, experiments, e.g., uniaxial tensile, are needed to be carried out for the purpose of obtaining the experimental force-displacement curve (i.e., target curve);
(b) Provide the learning samples of the GRNN model. The corresponding simulation models of the experiments are required to establish the same boundary and the initial conditions are considered. Next, several sets of material parameters (i.e., *C*_10_ and *C*_01_ for M-R model) will be predefined to produce different force-displacement curves. For the GRNN model, the sets of the material parameters can be taken as output vectors, and the corresponding force-displacement curves are input vectors. In this way, the learning samples of the GRNN model are given by FEA;
(c) Obtain the identified material parameters. Through the GRNN learning model, when the results of force-displacement calculated by FEA meet the requirements of accuracy, the corresponding output value at this moment is what we want.

### 3.3. An Example of GRNN-Based Approach Application

#### 3.3.1. Uniaxial Tensile Test with Hyperelastic Rubber Specimen

As mentioned in Section 3.2, in order to obtain the target value of the GRNN model, a uniaxial tensile test is constructed to measure the force-displacement curve of hyperelastic material. It should be noted here that the present GRNN-based approach is a kind of artificial intelligence method, and its architecture can be satisfied for both tensile and shear load conditions. In detail, no matter the tensile test or simple shear experiment [42], the experimental force-displacement curve can be obtained easily to provide the target value of the GRNN model. The experiment is implemented with silicone rubber (Hg6-678-74), which has been widely used in many fields, such as aviation, automobile, machinery, medicine and medical, etc. Silicone rubber has shown good low temperature resistance and can work at −55 °C. Meanwhile, silicone rubber is also outstanding in heat resistance, since it can work at 180 °C for a long time, and keep elastic at higher temperature (e.g., slightly higher than 200 °C) for a few weeks. In addition, silicone rubber also has good air permeability and its oxygen permeability is the highest among the synthetic polymers.

**Figure 2:**
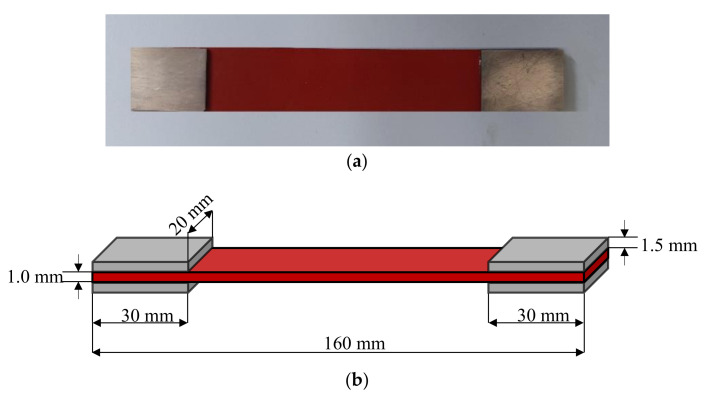
The specimen and its geometry employed in a uniaxial tensile test: (**a**) The image of silicone rubber specimen with bonded aluminum sheets; (**b**) The dimensions of silicone rubber specimen and aluminum sheets.

**Figure 3:**
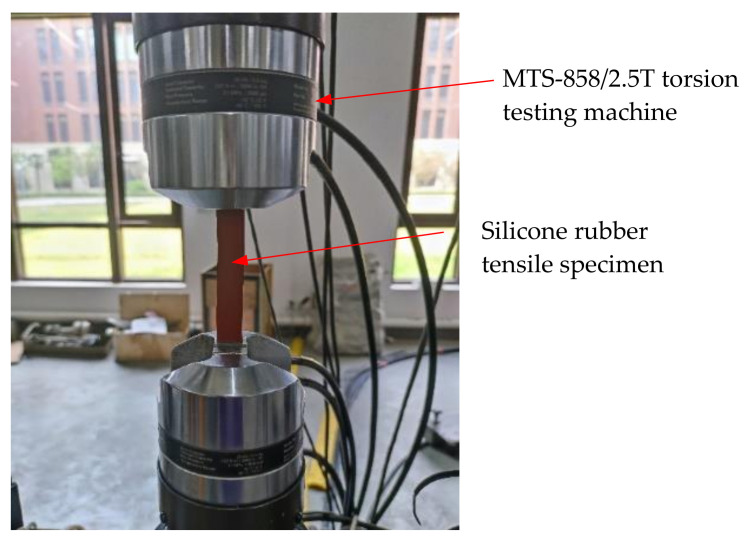
The experimental device of a uniaxial tensile test.

A strip specimen is adopted in the present uniaxial tensile test, as depicted in Figure 2. The geometry dimensions of the strip specimen are 160 mm × 20 mm × 1.0 mm, including 100 mm for the tensile test part, and 30 mm for the clamps at both ends of the specimen. Considering the thickness of the silicone rubber specimen is relatively thin, four aluminum sheets are bonded with 502 adhesive at the clamping region of tensile test machine to avoid local fracture at the clamping position, and the surface of the aluminum sheet is polished. All aluminum sheets have the same size, i.e., 30 mm × 20 mm × 1.5 mm. The test is carried out with MTS-858/2.5T torsion testing machine (SVL, Xi’an Jiaotong University) in air at room temperature, as shown in Figure 3. The test is performed in displacement-controlled mode with a constant loading rate of 20 mm/min.

**Figure 4:**
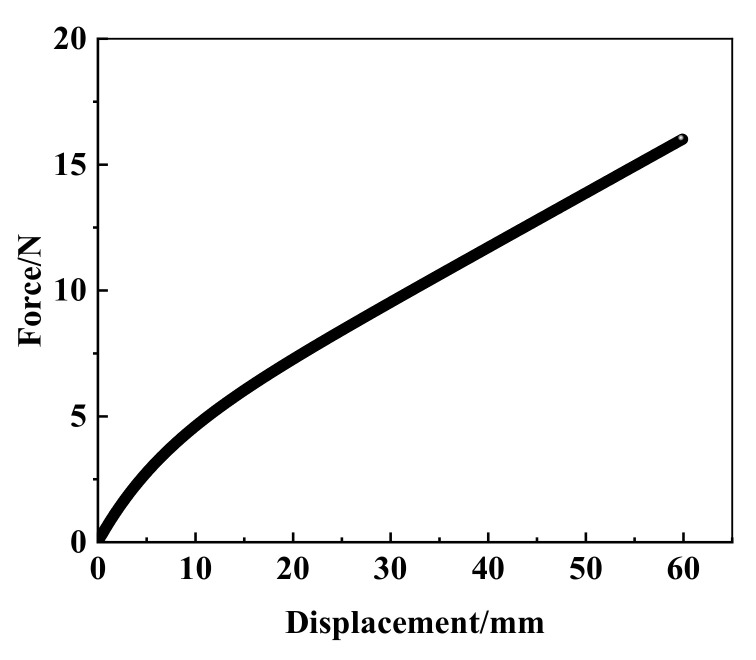
The experimental force-displacement curve of the uniaxial tensile test.

**Figure 5:**
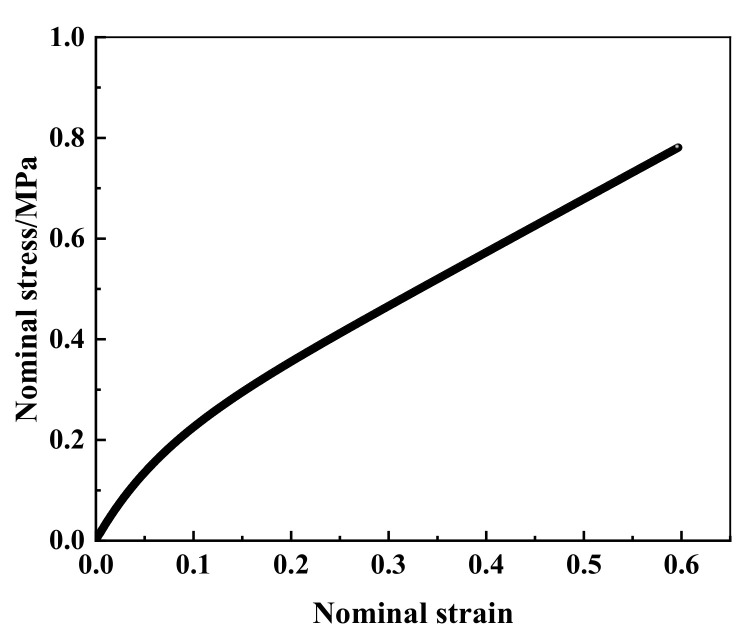
Nominal stress–strain curve under uniaxial tensile.

The force-load displacement curve collected by the experiment is presented in Figure 4. It can be seen that rubber shows obvious nonlinear characteristics. In detail, force varies linearly with the increasing displacement when the displacement is small, approximately 8 mm in present experiment, and the relation of force and displacement is in line with Hooke’s law. This is due to the rubber showing evident hyperelasticity during the large deformation. The slope of the force-displacement curve decreased slowly with the increase in displacement, and gradually tended toward a linear change again. The nominal stress–strain curve is obtained by processing the data of uniaxial tensile testing, as shown in Figure 5.

#### 3.3.2. FEA Calculation with Same Experimental Condition

**Figure 6:**
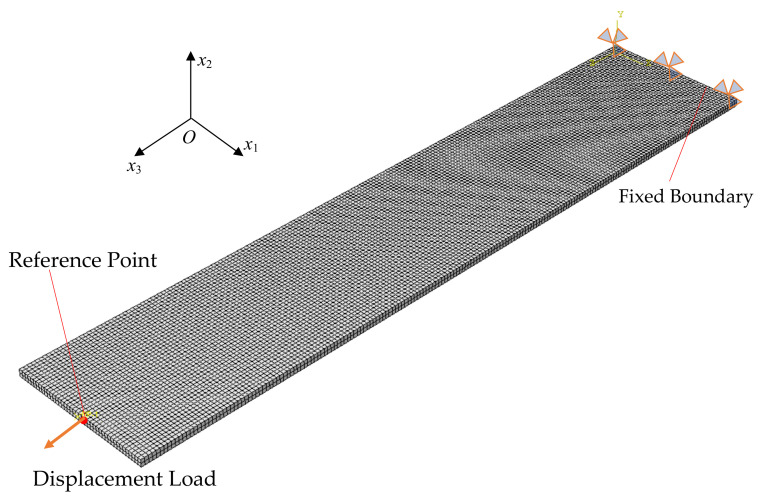
Finite element mesh and boundary conditions of silicone rubber specimen.

In this section, finite element simulation is carried out to obtain the learning samples of the GRNN model. Referring to the experimental conditions and geometry of the specimen, a numerical model is proposed. The silicone rubber specimen with bonded aluminum sheets is simplified to a rectangular geometry with a dimension of 100 mm × 20 mm × 1.0 mm. As depicted in Figure 6, one side of the strip specimen is fixed, and the other side is subjected to a uniaxial tensile displacement load of *u*_3_ = 20 mm over a time period of 60 s. The finite element mesh applied in this case consists of eight-node 3D stress elements with hybrid formulation (i.e., C3D8H). The mesh size is controlled by a global size of 0.5 mm. In special, a reference point is introduced to acquire the variations in force and displacement during the step time. So then, the displacement loading will be applied at the reference point, and transmitted through a reference point to the surface of the strip specimen.

**Figure 7:**
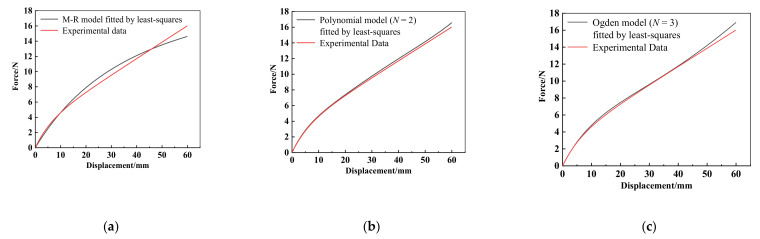
Results comparisons of: (**a**) M-R model; (**b**) polynomial model (*N*) = 2; and (**c**) Ogden model (*N* = 3) fitted by least-squares with experimental data.

In order to prepare the learning samples of GRNN, predefined material parameters are required to calculate several force-displacement curves. A widely used mathematical tool in data processing such as error estimation, system identification and prediction, i.e., least-squares fit, is built in software and can provide an initial set of model parameters, i.e., *C*_10_ = 0.0385 and *C*_01_ = 0.4052 for M-R model, *C*_10_ = −2.1506, *C*_01_ = 2.7355, *C*_20_ = 2.1308, *C*_11_ = −6.7135 and *C*_02_ = 6.3381 for polynomial model (*N* =2), *μ*_1_ = −3.9450, *α*_1_ = −2.3031, *μ*_2_ = −0.3774, *α*_2_ = −1.3540, *μ*_3_ = 5.4133 and *α*_3_ = −3.8436 for Ogden model (*N* = 3). Using these three sets of model parameters, the corresponding force-displacement curves are calculated and compared with the experimental force-displacement curve, as shown in Figure 7. It can found that there are significant differences between the least-squares fit result of the M–R model and experimental data when the deformation is larger than 10mm. For the polynomial model (*N* = 2), although the least-squares fit result is much better than the M–R model, the error becomes markedly more prominent as the deformation increases, and there is still room for improvement of accuracy. 

**Figure 8:**
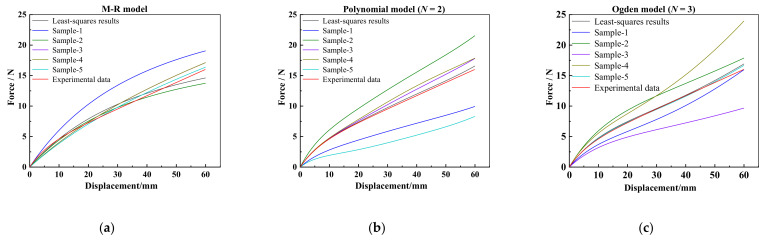
Numerical results of force-displacement curves and experimental curve: (**a**) M–R model results for learning samples of GRNN; (**b**) Polynomial model (*N* = 2) results for learning samples of GRNN; (**c**) Ogden model (*N* = 3) results for learning samples of GRNN.

Referring to the model parameters fitted by the least-squares method, the next five sets of model parameters, symbolled as sample-1, sample-2, sample-3, sample-4, and sample-5, are user-defined to calculate different force-displacement curves for the M–R model, the polynomial model (*N* = 2), and the Ogden model (*N* = 3), and finally six sets of model parameters are listed in Table 1. The numerical results of the corresponding force-displacement curves using the M-R model and polynomial model (*N* = 2) are plotted and compared with the experimental curve in Figure 8. Subsequently, the results of these force-displacement curves can be taken as input variables in GRNN learning, and the corresponding sets of parameters will be used as input vectors.

## 4. Results and Discussion

By substituting the different learning sample into the GRNN model implemented by the MATLAB code, and setting the accuracy requirement, the model parameters of the M–R model, the polynomial model (*N* = 2) and the Ogden model (*N* = 3) can be predicted through a series of sequential processing steps, including the cycle approximation of selection samples, FEA calculation, parameter optimization and GRNN-based learning. As listed in Table 2, the model parameters of the M–R model, the polynomial model (*N* = 2) and the Ogden model (*N* = 3) obtained from a GRNN-based approach and least-squares method are compared. In the following, the performances of a GRNN-based approach on parameter identification and optimization of the M–R model, the polynomial model (*N* = 2) and the Ogden model (*N* = 3) are discussed.

**Figure 9:**
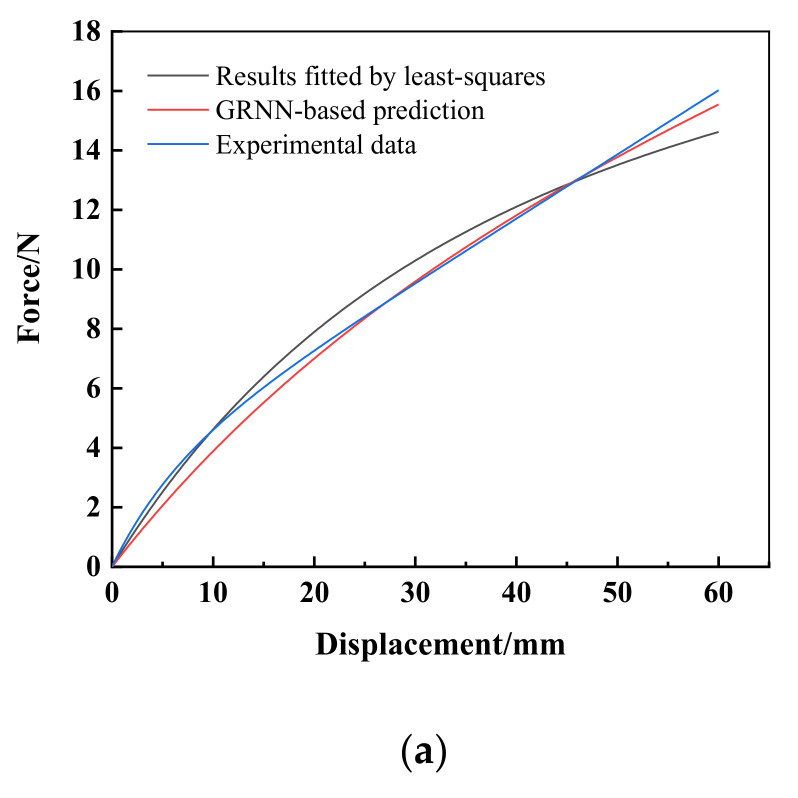

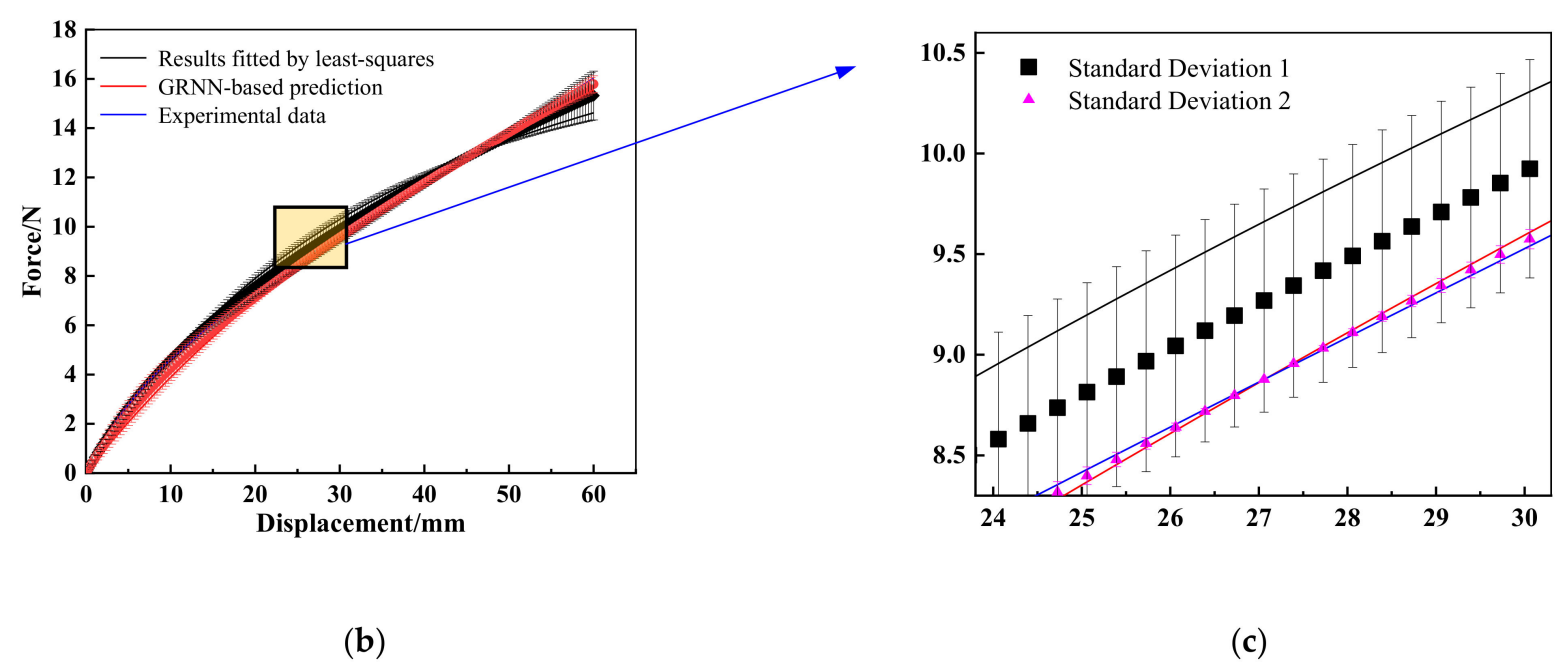
Results obtained by using M–R model.

(a) M–R model;

Using the different M–R model parameters predicted by a GRNN-based approach (red curve) and least-squares (black curve), the corresponding force-displacement curves are numerically calculated and compared with the experimental data (blue curves), as shown in Figure 9a. It can be seen that there are many interactions among blue, red and black curves, thus the comparison of results for GRNN, least-squares and experiment cannot be directly observed. In this subsection, the error bars represented standard deviations (see Figure 9b,c) and are firstly introduced to describe the accuracy of GRNN-based prediction. The length of the error bar corresponds to the value of the standard deviation, which means the difference between mean value and sample data.

Here, if the mean value of least-squares and the experimental data is defined as MV1, then, the differences between MV1 and force-displacement data fitted by least-squares, marked as standard deviation 1 (black square), can reflect the closeness of the least-squares results to the experimental data. Similarly, the values of standard deviation 2 (magenta triangle) represent the difference between MV2 (the mean values of GRNN-based prediction and experimental data) and GRNN-based prediction. The smaller the standard deviation (one or two) value, the closer the sample results (least-squares or GRNN-based prediction) and experimental data. Furthermore, from the partial enlargement presented in Figure 9c, it is clear to see that the lengths of the error bars associated with least-squares are obviously longer than those calculated by GRNN-based prediction, that is, the values of standard deviation 1 are larger than standard deviation 2, e.g., the maximum values of standard deviation 1 and 2 are 0.9912 and 0.5366, respectively. The results indicate that the force-displacement curve obtained from GRNN-based prediction is closer than those fitted by least-squares method to the experimental force-displacement curve. Additionally, we introduce the mean square error (the average value of the squared errors between the estimator variables, e.g., experimental data, and the variables being estimated, e.g., GRNN-based prediction or least-squares), and the mean absolute error [43] (the average distance between each experimental data and the GRNN-based prediction value or least-squares value) to evaluate the accuracy of the proposed method. The values of the mean square error and the mean absolute error of the GRNN-based prediction are 0.0215 and 0.1120, respectively. The results are obviously smaller than those of least-squares, i.e., 0.0721 (mean square error) and 0.1994 (mean absolute error). In other words, the results predicted by the GRNN-based approach can provide more precise M–R model parameters compared with those from the least-squares method, thus the material mechanical response can be analyzed more effectively. Therefore, it can be concluded that the GRNN-based approach is acceptable to be used as an effective method for parameter identification of the hyperelastic model.

**Figure 10:**
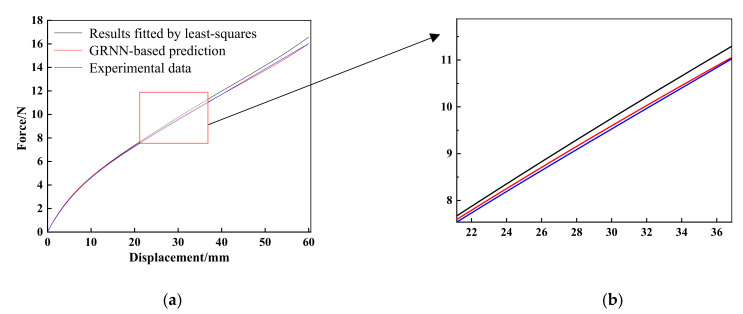
(**a**) Results obtained by using polynomial model (*N* = 2); (**b**) The partially enlarged views for specific deformation stages based on polynomial model (*N* = 2).

(b) Polynomial model (*N* = 2);

Similarly, the force-displacement curves associated with polynomial model (*N* = 2) obtained from least-squares (black curve), GRNN-based prediction (red curve) and experimental data (blue curve) are plotted in Figure 10. Intuitively, it is seen that the force-displacement curve calculated with GRNN-based prediction parameters is much closer to the experimental values than those resulted from least squares and is easy to analyze directly. In the present case, the corresponding values of the mean square error and mean absolute error for the GRNN-based approach are obtained as 0.5177 and 0.0954, which are less than those produced by least-squares, i.e., 0.6050 (mean square error) and 0.2385 (mean absolute error). It means that the GRNN-based prediction provides a better fit result than the simple fitting method, e.g., least-squares. For instance, the partially enlarged views of different deformation stages in Figure 10b are presented for different deformation stages. It can be concluded that the prediction results of the GRNN-based approach can maintain a relatively high accuracy for both small deformation and large deformation stages. In this model, the accuracy even increases with the displacement increasing within a certain range. In particular, from Figure 10, we can see the red curve (GRNN-based prediction) getting closer to the blue curve (experimental data) when the deformation varies from 8 to 40 mm.

**Figure 11:**
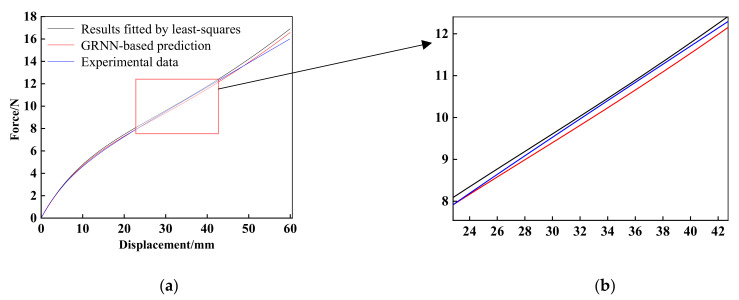
(**a**) Results obtained by using the Ogden model (*N* = 3); (**b**) The partially enlarged views for specific deformation stages based on Ogden model (*N* = 3).

(c) Ogden model (*N* = 3);

The results of force-displacement curves obtained by least-squares (black curve), GRNN-based prediction (red curve) and experimental data (blue curve) with Ogden model (*N* = 3) are presented for comparison in Figure 11. Similar to the results of the polynomial model (*N* = 2), the accuracy of the force-displacement curve of GRNN-based prediction can be directly observed and evaluated by the value of the mean square error and mean absolute error. For instance, the values of mean square error and mean absolute error of the GRNN-based approach are 0.0214 and 0.1120, while the mean square error and mean absolute error of least-squares are equal to 0.0722 (>0.0214) and 0.1994 (>0.1120), respectively. The results show that the proposed GRNN-based approach can work well in Ogden’s model. In addition, we would like to emphasize that the present method is a kind of machine-learning method; no matter whether the parameters of strain-energy density appear in a linear or nonlinear fashion, the architecture of the proposed GRNN-based approach is generic.

Based on the above discussion, it is indicated that the prediction of model parameters for the M–R model, the polynomial model (*N* = 2) and the Ogden model (*N* = 3) can be made more accurate with the GRNN-based approach. However, although all results calculated by the above three hyperelastic models can meet the accuracy requirements, the force-displacement curve obtained by the Ogden model is the closest to the experimental data, compared with those fitted by the M–R model and polynomial model (*N* = 2). Thus, the Ogden model (*N* = 3) is suggested to characterize the mechanical properties of the present silicone rubber.

## 5. Conclusions

Through a combination of experiment, numerical simulation, and GRNN, an effective and convenient GRNN-based approach for parameters identification of rubber-like hyperelastic material has been designed in the present study. In detail, the experiment is used to provide the target values of a GRNN model, the numerical model corresponding to experimental conditions is calculated to achieve the learning samples of a GRNN model, and the final identification of parameters is carried out in the main structure of GRNN. An example of parameters identification is performed by using uniaxial tensile testing of silicone rubber specimen. The results have shown that this GRNN-based approach can improve the accuracy and calculation efficiency of parameters identification for commonly used hyperelastic models, e.g., M–R model, polynomial model (*N* = 2), and is expected to be universal for other multi-parameter identification problems, e.g., the Ogden model (*N* = 3). The study implies that the GRNN-based approach is an excellent forecasting tool, which can predict and optimize the material parameters conveniently and automatically.

## Figures and Tables

**Table 1:** User-defined model parameters.

Model	Parameters	Least-Squares Method	Sample-1	Sample-2	Sample-3	Sample-4	Sample-5
M-R model	*C*_10_	0.0385	0.0510	0.0210	0.3160	0.2898	0.2898
	*C*_01_	0.4052	0.5270	0.4052	0.0420	0.0395	0.0455
Polynomial model (*N* = 2)	*C*_10_	−2.1506	−1.2904	−2.7958	−2.1506	−2.1506	−1.9506
	*C*_01_	2.7355	1.6413	3.5562	2.7355	2.7355	2.2535
	*C*_20_	2.1308	1.2785	2.7700	2.1308	2.1308	2.1308
	*C*_11_	−6.7135	−4.0281	−8.7276	−6.8000	−7.0000	−6.7135
	*C*_02_	6.3381	3.9029	8.2395	6.5000	6.8000	6.2530
Ogden model (*N* = 3)	*μ*_1_	−3.9450	−3.4560	−4.7340	−3.1560	−4.7340	−3.9513
	*α*_1_	−2.3031	−2.7637	−1.8425	−1.8425	−2.7637	−2.3068
	*μ*_2_	−0.3774	−0.3019	−0.4529	−0.4529	−0.4529	−0.3780
	*α*_2_	−1.3540	−1.6248	−1.0832	−1.6248	−1.6248	−1.3520
	*μ*_3_	5.4133	4.3306	6.4960	4.3306	6.4960	5.4057
	*α*_3_	−3.8436	−4.6123	−3.0749	−3.0749	−−4.6123	−3.8382

**Table 2:** Parameters obtained by GRNN-based approach and least-squares method.

Model	Parameters	GRNN-Based Approach	Least-Squares Method
M-R model	*C*_10_	0.2393	0.0385
	*C*_01_	0.1134	0.4025
Polynomial model (*N* = 2)	*C*_10_	−2.1505	−2.1506
	*C*_01_	2.7354	2.7355
	*C*_20_	2.1006	2.1308
	*C*_11_	−6.6185	−6.7135
	*C*_02_	6.2484	6.3381
Ogden model (*N* = 3)	*μ*_1_	−3.9516	−3.9450
	*α*_1_	−2.3069	−2.3031
	*μ*_2_	−0.3780	−0.3774
	*α*_2_	−1.3507	−1.3540
	*μ*_3_	5.4001	5.4133
	*α*_3_	−3.8342	−3.8436

## Data Availability

Not applicable.
